# Primary care experience and remission of type 2 diabetes: a population-based prospective cohort study

**DOI:** 10.1093/fampra/cmaa086

**Published:** 2020-09-12

**Authors:** Hajira Dambha-Miller, Alexander Day, Ann Louise Kinmonth, Simon J Griffin

**Affiliations:** 1 Primary Care Unit, Department of Public Health and Primary Care, University of Cambridge, Cambridge, UK; 2 MRC Epidemiology Unit, University of Cambridge, Cambridge, UK; 3 Division of Primary Care and Population Sciences, Faculty of Medicine, University of Southampton, Southampton, UK

**Keywords:** Doctor–patient relationship, epidemiology, patient experience, primary care, remission, Type 2 diabetes

## Abstract

**Background:**

Remission of Type 2 diabetes is achievable through dietary change and weight loss. In the UK, lifestyle advice and referrals to weight loss programmes predominantly occur in primary care where most Type 2 diabetes is managed.

**Objective:**

To quantify the association between primary care experience and remission of Type 2 diabetes over 5-year follow-up.

**Methods:**

A prospective cohort study of adults with Type 2 diabetes registered to 49 general practices in the East of England, UK. Participants were followed-up for 5 years and completed the Consultation and Relational Empathy measure (CARE) on diabetes-specific primary care experiences over the first year after diagnosis of the disease. Remission at 5-year follow-up was measured with HbA_1c_ levels. Univariable and multivariable logistic regression models were constructed to quantify the association between primary care experience and remission of diabetes.

**Results:**

Of 867 participants, 30% (257) achieved remission of Type 2 diabetes at 5 years. Six hundred twenty-eight had complete data at follow-up and were included in the analysis. Participants who reported higher CARE scores in the 12 months following diagnosis were more likely to achieve remission at 5 years in multivariable models; odds ratio = 1.03 (95% confidence interval = 1.01–1.05, *P* = 0.01).

**Conclusion:**

Primary care practitioners should pay greater attention to delivering optimal patient experiences alongside clinical management of the disease as this may contribute towards remission of Type 2 diabetes. Further work is needed to examine which aspects of the primary care experience might be optimized and how these could be operationalized.

**Key Messages**

• Thirty per cent of participants achieved remission of diabetes through lifestyle changes.
• Positive primary care experiences increased likelihood of remission.
• Clinicians must optimize patient experiences alongside biological markers.

## Background

Over 360 million primary care consultations occur each year in the UK accounting for 80% of all chronic disease management (1). Health care policy and a growing body of scientific evidence has highlighted the role of primary care as a valuable tool in slowing disease progress and managing complications associated with chronic disease (2,3). This has been emphasized in Type 2 diabetes, which affects 4 million people in the UK and accounts for one in every five primary care consultations. Previous trial and observational studies suggest a therapeutic effect of primary care consultation experiences on diabetes outcomes (4–6). We and others have carried out systematic reviews reporting that, where patients perceive better primary care experiences (i.e. those that involve listening, empathy and mutual decision-making), it may result in better intermediate diabetes outcomes, including lower HbA_1c_ levels (4–6). It is hypothesized that experiences, especially those focusing on the relational aspects, such as listening, empathy, understanding and shared decision-making, may result in enhanced trust and satisfaction, which, in turn, leads to be improved management. It may also empower and motivate patients towards adherence to medications and uptake of advice on physical activity or self-management of the condition.

To date, most evidence on the impact of primary care experience is limited to short-term follow-up (3,5). There is a paucity of evidence examining experiences in relation to longer term outcomes, including remission of the disease. Remission has been shown to be achievable in Type 2 diabetes and can be defined as an HbA_1c_ level of less than 6.5% (48 mmol/mol) in the absence of pharmacological or surgical intervention (7). Healthy behaviours, such as physical activity, dietary changes and subsequent weight loss, have been shown to induce remission (8–10). Advice on physical activity, dietary changes and initial discussions about referrals to weight loss or physical activity programmes commonly occur within primary care consultations. The contribution of the primary care experience to delivering effective lifestyle advice or initiating referrals in relation to weight loss services has been highlighted by the PROMISE modelling study, as well as larger trials of GP-led brief interventions in consultations (11,12). Given the position of primary care in providing an important context for diabetes management, including lifestyle advice and support, patient experiences of consultations could be an important determinant of remission. Accordingly, in this study, we describe the characteristics of people who achieve remission and aim to quantify the association between primary care consultation experience and remission of Type 2 diabetes over an extended follow-up of 5 years.

## Methods

### Study design and setting

We carried out a prospective cohort study using data collected from the Anglo-Danish-Dutch Study of Intensive Treatment in People with Screen Detected Diabetes in Primary Care (ADDITION) Cambridge (13). This was a cluster randomized controlled trial that examined the effects of intensive multifactorial treatment compared to routine care amongst individuals with screen-detected Type 2 diabetes (14). The trial was set in the East of England and used a stepwise screening programme to identify people who were at risk of Type 2 diabetes and then followed them up for 5 years where they received routine primary care (14,15). Type 2 diabetes was diagnosed according to the 1999 World Health Organization criteria (16) and participants were excluded at diagnosis if they were pregnant, breast-feeding, had psychiatric illness, prevented informed consent or had a disease with a likely prognosis of less than a year. At the start of the trial, there were 867 participants who agreed to participate across 49 GP practices. A detailed description of the trial has been reported in previous publications (17). The trial was not intended to alter consultation experiences and there were no significant differences in consultation measures between arms (17). Therefore, data were pooled from both arms and presented for the whole cohort.

### Measurements

At diagnosis, participants provided baseline information concerning age, sex and medication using standardized questionnaires. They also completed standardized questionnaires on self-reported age, gender, ethnicity (white, black, Asian, other or no response), socio-economic class and education level according to Register General Classifications.

Clinical and anthropometric measures, such as blood pressure, weight and height were undertaken by trained staff, following standard operating procedures (17,18). Further details on data collection methods have been reported previously (15,18).

Primary care experience was the main exposure variable. We specifically enquired about primary care experiences with GPs and nurses over the year following diagnosis of Type 2 diabetes. This was measured with a numerical score using the previously validated consultation and relational empathy (CARE) measure (19). The CARE measure quantifies experiences in primary care with a particular focus on relational aspects, such as empathy, compassion, understanding, shared decision-making and whether the patient felt listened to, considered as a whole person and understood (19,20). The CARE measure includes 10 questions, each answered on a Likert scale from 1 to 5, which are then summed together to give a total score. The CARE measure has been shown to have high reliability and validity when tested across thousands of primary care consultations in the UK (20). It has also been shown to have good predictive validity with a previous study demonstrating CARE measure results at baseline correlating strongly with CARE measure results many months later (21). The CARE measure asks about primary care experiences through the consultation and, to focus patient’s responses to diabetes-specific primary care experiences, the first line of the questionnaire read as follows: please rate the following statements about consultations related to Type 2 diabetes in the preceding year (20). The CARE measure then included questions shown in Box 1.

**Box 1: Sample questions from the CARE measure**

How good was the practitioner at:

1. making you feel at ease;
2. letting you tell your story;
3. really listening;
4. being interested in you as a whole person;
5. fully understanding your concerns;
6. showing care and compassion;
7. being positive;
8. explaining things clearly;
9. helping you to take control;
10. making a plan of action with you.

The main outcome was remission at 5-year follow-up determined by venous blood samples for HbA_1c_ levels. Remission was defined as an HbA_1c_ level on venous blood of <6.5% (48 mmol/mol) in the absence of any diabetes medications or surgery.

### Statistical analysis

Participant characteristics were summarized at baseline (i.e. at diagnosis) and 5-year follow-up using means [standard deviations (SDs)] or frequencies. To examine differences in characteristics between participants who achieved remission and those who did not, we used the chi-square and *t*-test where appropriate. Differences between characteristics of participants with and without missing data were also examined by comparing the distributions of factors measured at baseline between those who were and were not missing remission data. Data were pooled from both trial groups and presented for the whole cohort adjusted for trial group. We carried out a complete case analysis. Univariable logistic regression models were used to quantify the association between CARE score (as a continuous variable) and remission at 5 years, generating odds ratios and 95% confidence intervals. Multivariable models were then constructed adjusted on *a priori* reasoning. Stepwise nested models were examined as follows: Model 1 adjusted for baseline HbA_1c_, baseline weight and time since diagnosis; Model 2 additionally adjusted for socio-demographic characteristics, including age, sex, ethnicity (white or other), education level (full-time education finished at <16 or >16 years), occupation (managerial and professional, intermediate and manual), trial group and clustering by practice; and Model 3 additionally took into account disease severity and considered co-morbidities, including blood pressure, total cholesterol and all prescribed medications. Because, the starting point of HbA1c might be important in those who go into remission, all our models were adjusted for this at baseline, and we also carried out subgroup analysis, including only participants with HbA1c >6.5%. Statistical analysis was conducted in STATA version 15 (Stata, College Station, TX)

## Results

### Participant characteristics

Of the 867 ADDITION participants, there were 628 who had completed the CARE measure. Missing data was more likely amongst people who reported lower education levels or who had unskilled employment. Most of this cohort was white (97%) and male (68%) with a mean (SD) age of 60.8 (7.1) years. Forty-eight per cent of the cohort left school before 16 years and 42% reported unskilled backgrounds. Participant baseline socio-demographic characteristics are shown in Table 1 according to remission status at 5-year follow-up. Overall, 30% of this cohort achieved remission of diabetes at 5-year follow-up. From the original trial, 49.5% of these were in the routine care group and 51% were in the intervention group. We combined the groups and adjusted for trial group within our analyses. Those who achieved remission reported slightly higher education levels than those who did not achieve remission (45% of remitters completed education between 16 and 18 years compared to only 38% amongst non-remitters). Non-remitters were also more likely to be in unskilled professions compared to remitters (39% versus 44%). There were no other differences in baseline socio-demographic characteristics between remitters and non-remitters.

**Table 1.** Baseline participant socio-demographic characteristics amongst those who completed the CARE measure presented by remission status (*n* = 628)

Variables	Remitters		Non-remitters	
	*n*		*n*	
Mean (SD) age in years	191	61.1(6.9)	437	60.9(7.2)
Ethnicity (% white)	191	188(98)	437	421(96)
Male	191	112(59)	437	265(61)
Socio-economic class				
Professional	188	68(36)	425	145(34)
Skilled		47(25)		93(22)
Partly/not skilled		73(39)		187(44)
Education				
Full-time education finished at <16years	187	81(43)	429	219(51)
Full-time education finished at 16–18 years		85(45)		163(38)
Full-time education finished at >18years		21(11)		47(11)

Baseline data was collected between 2002 and 2006.

In terms of clinical baseline variables, remitters had better cardiovascular disease risk factor profiles with significant differences in baseline weight, HbA_1c_ levels, prescribed medications and number of co-morbidities. Between baseline and 5-year follow-up, clinical measures improved in both remitters and non-remitters; our observational findings show a significant improvements in weight, blood pressure, cholesterol and HbA_1c_ levels between baseline and 5-year follow-up. There were also significant increases in the number of participants prescribed blood pressure and lipid-lowering medication in both groups. These clinical measures amongst participants are summarized in Table 2. Primary care experiences according to the CARE measure did not vary significantly between remitters and non-remitters with a mean (SD) score of 39 (9.9) and 40 (9.3), respectively.

**Table 2.** Participant clinical characteristics at baseline and 5-year follow-up amongst those who completed the CARE score, presented by remission status at 5-year follow-up

Variables	Remitters				Non-remitters			
	*n*	Baseline	*n*	5 year	*n*	Baseline	*n*	5 year
Weight (kg)^a^	189	93.4 (17.8)	190	87.5 (17.5)	436	95.1 (17.9)	392	92.6 (18.4)
Systolic blood pressure (mmHg)^a^	191	142.3 (19.2)	191	133.5 (16.3)	435	141.6 (20.5)	393	135.2 (15.6)
Total cholesterol (mmol/l)^a^	184	5.3 (1.0)	191	4.1 (0.8)	431	5.4 (1.1)	387	4.1 (0.8)
HbA_1c_ %^a^ mmol/mol	185	6.7 (1.2) 50 (8.9)	191	6.1 (0.32) 43 (2.3)	429	7.6 (1.8) 60 (14.2)	385	7.5 (0.9) 58 (6.9)
Previous stroke	191	4 (2)	175	6 (3)	435	16 (3)	361	16 (4)
Previous myocardial infarct	190	9 (5)	175	9 (5)	432	36 (8)	367	35 (9)
Antihypertensive medication	191	105 (55)	191	144 (75)	437	254 (58)	437	323 (74)
Lipid lowering medication	191	38 (20)	191	154 (81)	437	100 (23)	437	335 (77)

Unless otherwise stated data are presented as *n* (%).

^a^Data are mean (SD).

### Remission of Type 2 diabetes at 5-year follow-up

In multivariate models, we observed that better primary care experiences (according to unit changes in CARE measure) were associated with statistically higher odds of remission. In the maximally adjusted model, this was associated with 3% higher odds of remission with each unit increase in CARE measure. Similar positive trends were observed in unadjusted models, but these did not reach significance. These results are summarized in Table 3 below. We also conducted subgroup analysis, including only those with HbA_1c_ levels >6.5% and we found similar statistically significant results.

**Table 3.** Associations between primary care consultation experience over the first year and the odds of remission at 5-year follow-up

	*n*	Odds ratio	95% CI		*P*-value
Univariable model	628	1.01	0.99	1.03	0.16
Model 1	603	1.02	1.00	1.04	0.05
Model 2	599	1.02	1.00	1.04	0.04
Model 3	545	1.03	1.01	1.05	0.01

Model 1 adjusted for baseline HbA_1c_, baseline weight and time since diagnosis. Model 2 additionally adjusted for age, sex, ethnicity, education level, occupation, trial group and clustering by GP practice. Model 3 additionally considered baseline blood pressure, total cholesterol and medication use.

## Discussion

To the best of our knowledge, this is the first study to examine the primary care experience in relation to remission of Type 2 diabetes. Our results demonstrate a modest but important potential of the primary care experience in achieving remission of Type 2 diabetes.

### Comparison to existing literature

Our study adds to the growing body of evidence and governmental policy for ‘making every consultation count’ towards preventing chronic disease and its associated complications (2,22). Until now, the evidence to support this in Type 2 diabetes has mainly looked at short-term intermediate outcomes (4–6,23). We now report that remission over the longer term is achievable within a population-based sample across many GP practices representing a range of experiences of primary care. Our observational findings show a statistically positive association, but the odds of remission were only increased by 1–3% with better reported experiences. This is a 3% higher odds of remission with each unit increase in CARE measure. Clinically, this suggests that the patient’s perception of experience alone is important but may only contribute a small part of what is likely to be a complex and multifactorial process of achieving remission.

Our results suggest that weight loss is far more important on this pathway (9,24,25). We observed significant changes in the weight between baseline and 5-year follow-up corresponding to high rates of remission and have published this data in a separate paper (26). Primary care experiences that were perceived positively by patients may well be those that included supportive and empowering discussions about weight loss and subsequent adherence to doctor’s advice. Previous evidence in UK primary care cohorts suggest that opportunistic discussions about weight loss should occur more frequently as they can lead to adherence to weight loss activities (27). Our study did not contain data on the specific content of these consultations or which aspects of the experience were most meaningful to patients.

In our results, we also reported differences between remitters and non-remitters in prescribed medications. Remission includes participants who are not prescribed hypoglycaemic medication but there were differences in lipid-lowering and blood pressure medications. Steinke *et al.* previously demonstrated in non-diabetes populations that communication and the relational aspects of the experience with practitioners were significant contributors to the practitioner’s decision to prescribe medications (28). Similarly, Bradley’s qualitative study with 69 GPs and 5 GP trainees suggested that decisions to prescribe medications were influenced by the doctor–patient relationship (29). Earlier studies show that patient experience measures tend to be more positive if medications were prescribed (30). It is plausible, therefore, that patient’s rating of primary care experiences may reflect the doctor–patient relationship and subsequent decisions by practitioners to prescribe medications. Others have demonstrated associations between blood glucose and both statins and beta blockers, although the direction of these proposed mechanisms does not explain our findings towards remission (31). Although generic medications were considered in our statistical models, they were not specific to these drugs, so we were unable to explore these further. Furthermore, multimorbidity and continuity of care are likely to be important factors in patient’s perceptions of consultations. People with multiple health conditions are likely to see their health practitioners more frequently, and continuity of care has been associated with better patient satisfaction (32,33). Although these were not examined within our study, they are likely to be important factors in perceptions of care given that most people with Type 2 diabetes have three or more co-morbidities.

### Strengths and weakness

This is the first study to consider remission of Type 2 diabetes in relation to primary care experience. The follow-up period of 5 years with a low attrition rate is a clear strength along with the measures used (8,9). The CARE measure has undergone extensive validity and reliability testing around the UK and has been shown to be meaningful and relevant to patients and their practitioners (20). Our participants include an extensive geographical area in the East of England with a heterogeneous sample of practices. There is also heterogeneity in our sample population in terms of socio-economic groups and disease severity. However, our geographical location in East Anglia under-represents ethnic minorities who are more prevalent in other parts of the UK. Burt *et al.* have suggested that primary care experiences vary considerably by ethnic group and, thus, our findings may not be generalizable to all diabetes populations (34). It is possible that the personalized patient-centred care approach that is investigated in our study might be effective for specific socio-economic and ethnic groups, but minorities and deprived groups are often under-represented in research, including within our study.

Compared to other studies, our follow-up and remission rate was high (8,35). This may reflect a highly engaged and responsive cohort. Previous studies on remission are not directly comparable as they are all shorter in duration and include intensive dietary or physical activity requirements, such as intensive low-calorie diet of 624–700 kcal/day for 8 weeks or an intensive diet replacement of 825–853 kcal/day through a formula diet for 3–5 months (24). We followed-up participants for 5 years after an intervention for screening that had with no requirements for intensive physical activity or dietary changes. Missing data and/or sample attrition can introduce a selection bias in longitudinal cohorts. Studies on primary care cohorts with comparable participants in terms of age and sex have shown that this is not necessarily inevitable (36).

Other limitations are that we captured primary care experience at a single time point through a consultation over the first year after new diagnosis. Experiences may vary over time, especially, as chronic disease progresses and health care systems change. However, the CARE measure has been shown to have good durability and predictive value (21). Our study does not capture relational continuity of care over the course of the disease. Although evidence of the effect of continuity of care on health outcomes is mixed, it is possible that patients who saw the same practitioner over time may well have been more likely to remit (37). We also relied on self-report measures of experiences, which may include a recall bias (38). Our work would have been strengthened by the inclusion of objective measures such as video or audio recording of the experiences to capture data on discussions around weight loss, physical activity or referrals that could contribute towards the behaviours needed to achieve remission.

We carried out a complete case analysis, so those with missing CARE measures were not included. These participants may have been those with poor primary care experiences who had not returned to see their practitioners and not achieved remission. It is also possible that our findings are due to chance. Confounding may be another explanation for our findings, which means that positive patients who are optimistic in their outlook and, thus, their behaviours, may respond more positively to the questionnaire (39). Finally, the social context is important to consider as our findings as we observed that those who achieved remission reported more educational opportunity than non-remitters. Our findings may, therefore, simply reflect greater resources to health care and lifestyle amongst those who are more educated rather than experiences of primary care.

## Conclusion

Primary care remains an important context for the delivery of health care in the UK. Optimal patient experiences could be linked to remission of Type 2 diabetes, emphasizing the need for clinicians to pay adequate attention to these aspects of care. Our study with a population-based cohort in routine primary care shows that successful remission through weight loss was not related to over-prescribing or intensive intervention. Instead, we highlight the important role of the patient–practitioner relationship; this is a core aspect of general practice. Health care policy that supports and encourages the provision of person-centred, integral and continuous primary care could have important clinical effects across health care conditions. Future research is needed to replicate these findings on larger and more diverse populations, including ethnic minorities and those from more deprived socio-economic backgrounds. Further work will need to capture more information on the consultation experience content, including discussions about weight loss, lifestyle changes and referral to exercise or diet programmes. This could help to unpick the mechanisms underlying the observed associations.

## Declarations

Funding: ADDITION-Cambridge was supported by the Wellcome Trust (grant reference no: G061895), the Medical Research Council (grant reference no: G0001164 and Epidemiology Unit programme: MC_UU_12015/4), the NIHR Health Technology Assessment Programme (grant reference no: 08/116/300), NIHR Programme Grants for Applied Research (RP-PG-0606-1259) National Health Service R&D support funding (including the Primary Care Research and Diabetes Research Networks) and the NIHR. SJG is an NIHR Senior Investigator. The University of Cambridge has received salary support in respect of SJG from the NHS in the East of England through the Clinical Academic Reserve. Bio-Rad provided equipment for HbA1c testing during the screening phase. The Primary Care Unit is a member of the NIHR School for Primary Care Research and supported by NIHR Research funds. HDM was an NIHR Doctoral Research Fellow at the time of this study and is now an NIHR Clinical Lecturer. The views expressed are those of the author(s) and not necessarily those of the NHS, the NIHR or the Department of Health and Social Care.

Ethics approval: participants gave written informed consent and the study was approved by an Ethics Committee (Eastern MREC, reference 02/5/54 and ADDITION 5 Year: Cambridgeshire 1 REC, reference 08/H0304/67). The ADDITION-Cambridge trial is registered as ISRCTN86769081.

Data availability: No data available.
